# Supplementary material for: Analysis of the Curative Effect of Temporomandibular Joint Disc Release and Fixation Combined with Chitosan Injection in the Treatment of Temporomandibular Joint Osteoarthrosis
Source: J Clin Med. 2023 Feb 19;12(4):1657. doi: 10.3390/jcm12041657 (PMC9966182; doi:10.3390/jcm12041657)
Supplement: Supplementary file 1 [file jcm-12-01657-s001.zip › jcm-2160574-supplementary.pdf]

Supplementary Materials:

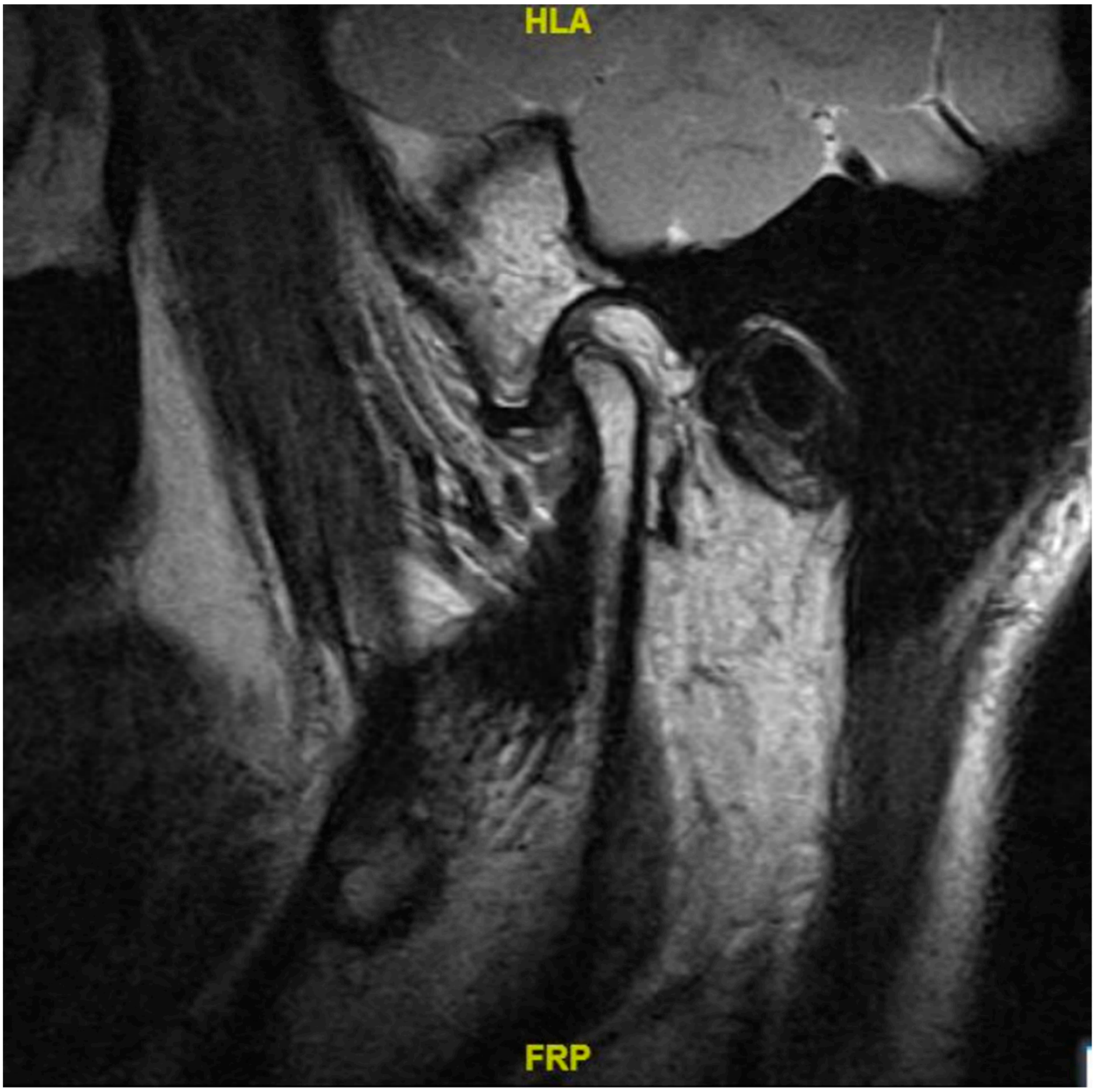

Figure S1. TMJ MRI revealed bone destruction pretreatment and disc displacement without reduction.

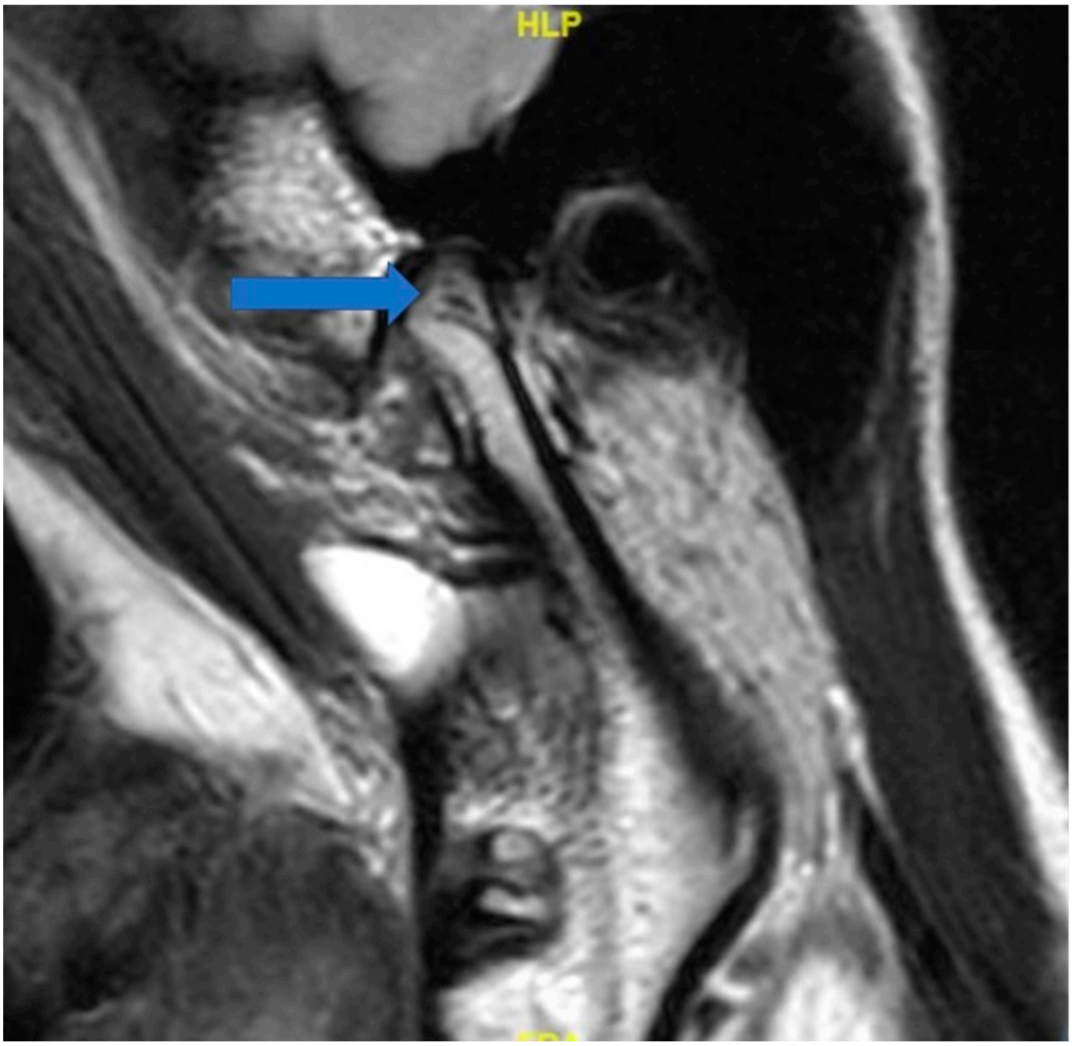

Figure S2. New bone(blue arrow) was found by MRI 6 months after surgery and the disc is reduced to normal position by surgery.
